# Change in network connectivity during fictive-gasping generation in hypoxia: prevention by a metabolic intermediate

**DOI:** 10.3389/fphys.2014.00265

**Published:** 2014-07-23

**Authors:** Andrés Nieto-Posadas, Ernesto Flores-Martínez, Jonathan-Julio Lorea-Hernández, Ana-Julia Rivera-Angulo, Jesús-Esteban Pérez-Ortega, José Bargas, Fernando Peña-Ortega

**Affiliations:** ^1^Departamento de Neurobiología del Desarrollo y Neurofisiología, Instituto de Neurobiología, Universidad Nacional Autónoma de MéxicoQuerétaro, México; ^2^División de Neurociencias, Instituto de Fisiología Celular, Universidad Nacional Autónoma de MéxicoMéxico D.F., México

**Keywords:** pre-Bötzinger complex, gasping, SIDS, Kreb's cycle, network analysis

## Abstract

The neuronal circuit in charge of generating the respiratory rhythms, localized in the pre-Bötzinger complex (preBötC), is configured to produce fictive-eupnea during normoxia and reconfigures to produce fictive-gasping during hypoxic conditions *in vitro*. The mechanisms involved in such reconfiguration have been extensively investigated by cell-focused studies, but the actual changes at the network level remain elusive. Since a failure to generate gasping has been linked to Sudden Infant Death Syndrome (SIDS), the study of gasping generation and pharmacological approaches to promote it may have clinical relevance. Here, we study the changes in network dynamics and circuit reconfiguration that occur during the transition to fictive-gasping generation in the brainstem slice preparation by recording the preBötC with multi-electrode arrays and assessing correlated firing among respiratory neurons or clusters of respiratory neurons (multiunits). We studied whether the respiratory network reconfiguration in hypoxia involves changes in either the number of active respiratory elements, the number of functional connections among elements, or the strength of these connections. Moreover, we tested the influence of isocitrate, a Krebs cycle intermediate that has recently been shown to promote breathing, on the configuration of the preBötC circuit during normoxia and on its reconfiguration during hypoxia. We found that, in contrast to previous suggestions based on cell-focused studies, the number and the overall activity of respiratory neurons change only slightly during hypoxia. However, hypoxia induces a reduction in the strength of functional connectivity within the circuit without reducing the number of connections. Isocitrate prevented this reduction during hypoxia while increasing the strength of network connectivity. In conclusion, we provide an overview of the configuration of the respiratory network under control conditions and how it is reconfigured during fictive-gasping. Additionally, our data support the use of isocitrate to favor respiratory rhythm generation under normoxia and to prevent some of the changes in the respiratory network under hypoxic conditions.

## Introduction

Neuronal assemblies are embedded in complex networks that act in concert to generate the function of a given brain region (Lindsey et al., 2000; Ramirez et al., 2004, 2007; Carrillo-Reid et al., 2008; Segers et al., 2008; Galan et al., 2010; Jaidar et al., 2010; Ott et al., 2011). Several neural circuits produce spontaneous synchronous activity through the interactions of the intrinsic properties of their neurons and the chemical and electrical synapses that link them into assemblies and networks (Ramirez et al., 2004; Carrillo-Reid et al., 2008; Peña, 2009; Jaidar et al., 2010; Zavala-Tecuapetla et al., 2014). Neuronal assembly bursting is an important feature that ensures the reliability of synaptic transmission, plasticity, and information processing (Lisman, 1997). It induces the activity of central pattern generators (CPGs), which are responsible for vital functions such as breathing (Ramirez et al., 2004, 2013; Peña, 2009), whose CPG is located in the preBötzinger Complex (preBötC; Smith et al., 1991). Although some insights about the respiratory network configurations required to produce different patterns of breathing activity have been revealed by extensive cell-focused studies (St. John and Bianchi, 1985; Richter et al., 1991; Ballanyi et al., 1994; England et al., 1995; Ramirez et al., 1998; St. John, 1999; Lieske et al., 2000; Thoby-Brisson and Ramirez, 2000; Peña et al., 2004; Paton et al., 2006; Zavala-Tecuapetla et al., 2008; Lalley and Mifflin, 2012; Ramírez-Jarquín et al., 2012), a more detailed description of respiratory circuit configurations is emerging from structural imaging (Hartelt et al., 2008; Mironov, 2009) and the evaluation of cell assemblies while maintaining single-cell resolution using dynamic calcium imaging (Okada et al., 2012; Gourévitch and Mellen, 2014) or multielectrode arrays (MEAs; Segers et al., 2008; Galan et al., 2010; Morris et al., 2010; Ott et al., 2011; Carroll and Ramirez, 2013; Carroll et al., 2013). These techniques allow functional network analysis and may reveal distinct configurations of neural circuits in control conditions or under various physiological and/or pathological conditions (Ramirez et al., 2004; Carrillo-Reid et al., 2008; Mironov, 2009; Jaidar et al., 2010; Peña et al., 2010).

The preBötC is able to adjust its function to fit different metabolic demands by acquiring different configurations (Richter et al., 1991; Ballanyi et al., 1994; England et al., 1995; Ramirez et al., 1998; Peña, 2009; Rivera-Angulo and Peña-Ortega, 2014). The network focused studies of the respiratory network have started to reveal that the preBötC forms dense clusters of respiratory cells with occasional connections between them (Hartelt et al., 2008; Mironov, 2009; Gaiteri and Rubin, 2011) that can be reconfigured in a cycle-by-cycle manner (Carroll et al., 2013; Carroll and Ramirez, 2013; Koshiya et al., 2014). Modeling based on this evidence indicates that the activity of the preBötC is highly dependent on its circuit configurations, the intrinsic dynamics of neurons at central network positions, and the strength of synaptic connections between neurons (Gaiteri and Rubin, 2011). These results suggest that the study of circuit configurations and reconfigurations is key to understand the flexibility of breathing generation during different metabolic states (Mironov, 2009; Galan et al., 2010). One of the extreme examples of respiratory network flexibility is the reconfiguration process that this network undergoes under extreme hypoxic conditions (Lieske et al., 2000; Peña and Ramirez, 2005; Rivera-Angulo and Peña-Ortega, 2014): Lieske et al. (2000) documented that this process allows the preBötC to change its burst pattern from fictive-eupnea in normoxia to fictive-gasping in hypoxia. Since the changes in the burst pattern as well as in firing patterns of respiratory neurons observed in the brainstem slice preparation closely resemble those observed *in vivo* during the transition from actual eupnea to actual gasping, we adopted the term “fictive” to refer to those patterns generated by the preBötC *in vitro* (Lieske et al., 2000; Lieske and Ramirez, 2003). Extensive cell-focused studies revealed some of the changes in both the intrinsic and synaptic properties of the respiratory neurons involved in this transition (Richter et al., 1991; Ballanyi et al., 1994; Ramirez et al., 1998; Lieske et al., 2000; Thoby-Brisson and Ramirez, 2000; Peña et al., 2004; Paton et al., 2006; Zavala-Tecuapetla et al., 2008; Ramírez-Jarquín et al., 2012). However, the main changes in circuit configuration when passing from normoxia to hypoxia have not been described. With the help of multi-electrode array (MEA) recordings (Lindsey et al., 2000; Segers et al., 2008; Galan et al., 2010; Ott et al., 2011; Carroll and Ramirez, 2013; Carroll et al., 2013), we wanted to establish whether the respiratory network reconfiguration in hypoxia involves changes in the number of active respiratory elements, in the functional connections among the elements, or in the strength of these functional connections. The other aim of this study was to characterize the changes in respiratory network configuration upon the application of a citric acid cycle intermediate (isocitrate), particularly during fictive-gasping generation (Rivera-Angulo and Peña-Ortega, 2014). Since a failure to generate gasping has been linked to Sudden Infant Death Syndrome (SIDS; Lijowska et al., 1985; Poets et al., 1999; Peña and García, 2006; Peña, 2009), the study of gasping generation, as well as the identification of pharmacological approaches to promote it, may have clinical relevance as a preventive intervention in babies at risk for SIDS (Peña and García, 2006; Peña, 2009).

We have recently shown that supplementation of the respiratory network with the metabolic intermediate isocitrate increases preBötC activity in normoxia and favors gasping generation in hypoxia both *in vitro* and *in vivo* (Rivera-Angulo and Peña-Ortega, 2014). Several metabolic intermediates can modulate both the intrinsic and the synaptic properties (Dehaven and Carpenter, 1964; Böhmer et al., 1976; Chaplain et al., 1976; Dinse et al., 1976; Shoji, 1992) of neurons in different networks, including those in the respiratory network (Dehaven and Carpenter, 1964; Böhmer et al., 1976; Chaplain et al., 1976; Dinse et al., 1976), therefore we hypothesized that isocitrate could influence respiratory network configuration and also modulate its reconfiguration in hypoxia.

We analyzed neuronal activity recorded by two types of respiratory recording elements: those arising from single neurons (unitary activity) and those arising from groups of neurons [multiunitary activity (MUA)] (Kirkwood, 1979; Kashiwagi et al., 1993; Shen et al., 2002; Li et al., 2003; Eugenin et al., 2006; Segers et al., 2008; Ott et al., 2011; Lalley and Mifflin, 2012; Road et al., 2013). We used the correlated firing among respiratory elements as an assessment of “functional connectivity,” and the correlation value was assumed to be proportional to the connectivity strength (Kirkwood, 1979; Kashiwagi et al., 1993; Shen et al., 2002; Li et al., 2003; Eugenin et al., 2006; Segers et al., 2008; Ott et al., 2011; Lalley and Mifflin, 2012; Road et al., 2013). We did not investigate how many of these functional connections are formed by real synaptic connections and how many of them were synchronized by other elements of the net. Finally, we built correlation linkage maps in order to illustrate and compare functional configurations of the respiratory circuit in the different experimental conditions (Segers et al., 2008; Ott et al., 2011). Surprisingly, and in contrast with previous suggestions from cell-focused studies (Peña, 2009), our results show that during the transition to fictive-gasping generation, the number of active respiratory elements, their activity (firing frequency), and the number of their functional links do not change dramatically. In contrast, the main change in the reconfiguration of the respiratory network during hypoxia involved a complex modification of the amount of correlated activity between the elements of the circuit, suggesting a global reduction in the strength of network interactions. Interestingly, isocitrate prevents these changes in the strength of circuit connectivity under hypoxic conditions.

## Materials and methods

### Animals

Experiments were performed using 6–9-day-old (P6–P9) CD-1 mice (*N* = 12). All experimental protocols were approved by the local Committee on Ethics of Animal Experimentation (INB-UNAM). Experiments were performed according to the Mexican Official Norm for the Use and Care of Laboratory Animals (NOM-062-ZOO-1999).

### Brainstem slice preparation

**Figure 1:**
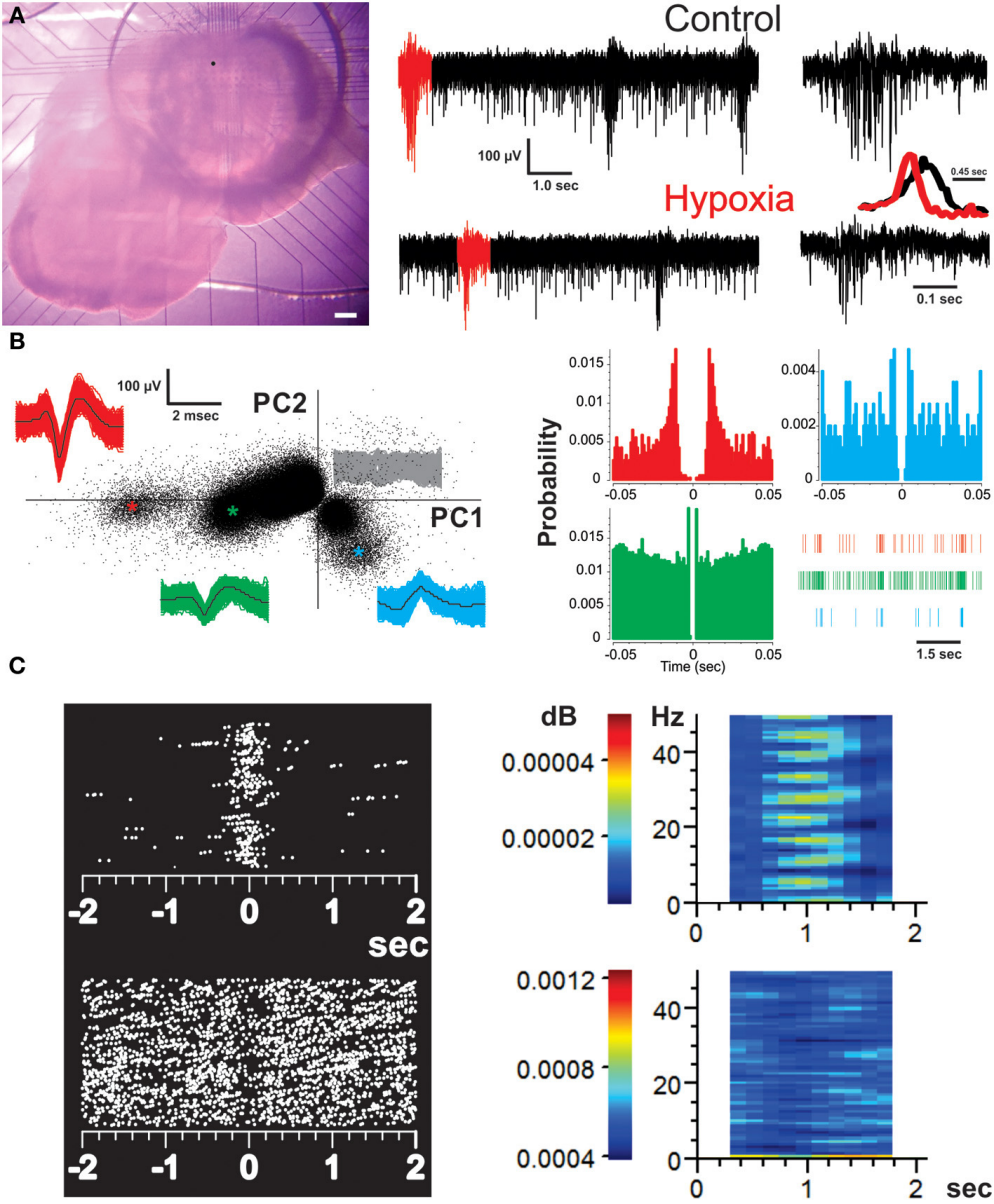
**Recording of the respiratory network with multielectrode arrays (MEAs) and identification of respiratory units**. **(A)** Micrograph of a brainstem slice preparation on top of a MEA that covers the area of the ventral respiratory group (including the preBötC). Scale bar represents 200 μm. The dot on the slice represents the location of the MEA electrode from which the recordings represented on the right were taken. The recordings from the chosen electrode show spontaneous activity of the preBötC in normoxic (control) and hypoxic conditions. The bursts in red were expanded on the right to better appreciate the difference in burst pattern between fictive-eupnea (control) and fictive-gasping (hypoxia). The inset shows the averaged peak-to-peak amplitude for 30 bursts in normoxia (black) and hypoxia (red). Note the reduction in burst duration and rise-time in those bursts generated in hypoxia. **(B)** Spike sorting analysis of the chosen electrode to discriminate individual respiratory units recorded from it. The graph on the left is a two dimensional feature space spanned by the weights of the first and second principal components (PC1 and 2). Each point indicates one threshold-crossing event (2.6 *SD* of noise). The insets show all spike waveforms (and their mean in black) obtained from the individual clusters identified by colors (as ^*^ in the PC plot). The signals belonging to the noise (central clusters) are represented in gray. The corresponding auto-correlograms on the right (color-coded as on the left) display clear refractory periods. A raster of each unit in a given time is provided. Each vertical line represents a spike. **(C)** Perievent raster plots (left) and perievent spectrograms (right) of representative inspiratory (top) and expiratory (bottom) neurons. Each dot represents a spike. Color bars in the spectrograms represent the power value.

Details of the slice preparation have been previously reported (Peña et al., 2004, 2008). Briefly, animals were anesthetized and decapitated, and the brainstem was quickly removed and placed in ice-cold artificial cerebrospinal fluid (ACSF) constantly bubbled with carbogen (95% O2 and 5% CO2). The ACSF contained (in mM) 119 NaCl, 3 KCl, 1.5 CaCl2, 1 MgCl2, 25 NaHCO3, and 30 D-glucose (pH 7.4). The brainstem was glued rostral-end upward onto an agar block, mounted on a vibratome (Vibratome Company, St. Louis, MO), and serially sliced until the rostral boundary of the preBötC was identified using anatomical landmarks such as the disappearance of the facial nucleus and appearance of the inferior olive as well as the ambiguous and hypoglossal nuclei (Figure 1A). A single slice (550 μm thick; Peña and Ramirez, 2002) per animal was obtained and transferred into a recording chamber with a total volume of 2 ml and containing a MEA at the bottom (Multi-Channel Systems; Reutlingen, Germany; Figure 1A). There, the slice was continuously perfused by recirculating 200 ml ACSF at a flow rate of 10 ml/min and constantly bubbled with carbogen to ensure efficient oxygenation and exchange of the solution (Peña et al., 2004, 2008; Zavala-Tecuapetla et al., 2008, 2014). A temperature controller (Multi-Channel Systems; Reutlingen, Germany) maintained the temperature at 30 ± 1°C. To allow long-term recordings of rhythmic population activity, extracellular KCl was elevated from 3 to 8 mM over a span of 75 min before starting the recordings (Tryba et al., 2003; Figure 1A). Hypoxic conditions were induced by removing carbogen and bubbling the ACSF for 15 min with 95% N2 and 5% CO2 (Peña et al., 2004, 2008 Figure 1A). Our experimental conditions are almost identical to those used and characterized by Hill et al. (2011). When ACSF was bubbled with carbogen under these conditions, the PO2 in the recording chamber was 679 ± 30 Torr, while at a depth of 300 mμ within the slices, PO2 was 58 ± 16 Torr, which can be considered normoxic conditions (Hill et al., 2011). In contrast, when ACSF was bubbled with 95% N2 and 5% CO2, the PO2 in the recording chamber was 38 ± 28 Torr, while 300 μm deep in the slices PO2 was 5 ± 6 Torr, which can be considered hypoxic (Hill et al., 2011). Isocitrate (Sigma-Aldrich, St. Louis, MO) was bath applied at a final concentration of 3 mM for 1 h, as previously reported using the same preparation (Hülsmann et al., 2000; Rivera-Angulo and Peña-Ortega, 2014). Fresh, 1000× isocitrate stock solution was prepared by dissolving it in distilled water. The last 10 min of isocitrate application or hypoxia, along with 10 min of control, were used for the analysis (Rivera-Angulo and Peña-Ortega, 2014).

### Recordings

Multisite extracellular recordings were performed using the MEA2100-system (Multi-Channel Systems, Reutlingen, Germany; Figure 1A). A 60-electrode array (TiN/SiN) is composed of a 6 × 10 grid with 100-μm inter-electrode spacing and 30-μm electrode diameter (Figure 1A). Data acquisition was controlled by MC_Rack software (Multi-Channel Systems Reutlingen, Germany). Raw data was digitized at 25 kHz and stored in a personal computer for off-line analysis.

### Analysis

**Figure 2:**
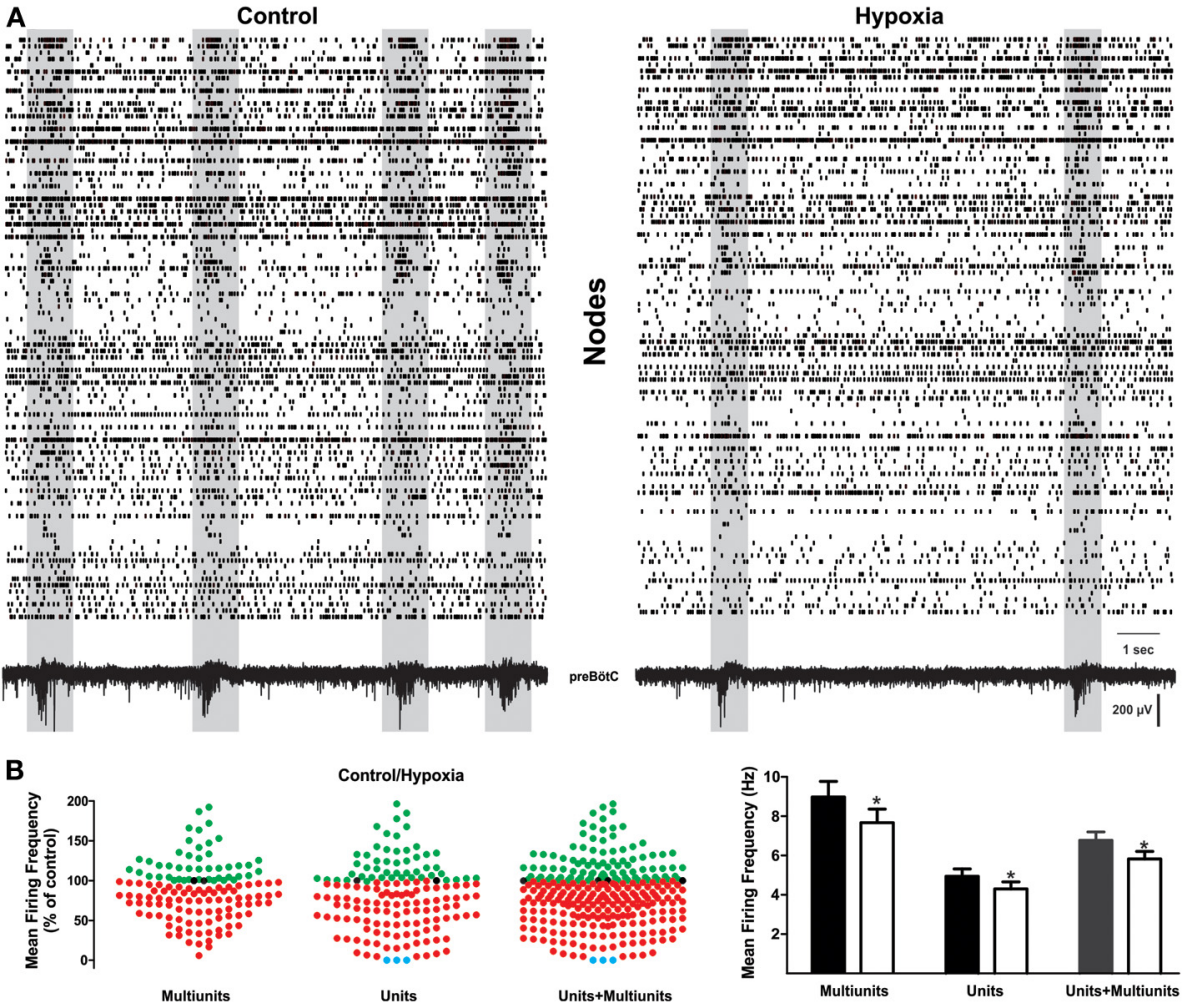
**Change in respiratory network activity from normoxic to hypoxic conditions. (A)** Raster plots of action potential recordings on different electrodes of the MEA. Lines may represent unitary or multi-unitary recordings, sometimes recorded with the same electrode when these could be distinguished. Each row is the activity obtained from a respiratory element and each vertical line represents a spike. Both fictive-eupnea (control; left) and fictive-gasping (hypoxia; right) are shown. Shadowed frames denote time windows containing an inspiratory event, as depicted by the recording at the bottom. It is readily apparent that these time windows include a significant enhancement of correlated firing among different elements, and they significantly decrease during hypoxia. **(B)** Quantification of normalized activity change in all respiratory recording elements during hypoxic conditions as compared to control conditions (set as 100%; *n* = 5 slices from different animals). Left, shows the change in firing frequency in each row (element) of the raster plots for multi-unitary, unitary recordings, and the merge of both. Recording elements exhibiting an increase in activity during hypoxia are represented as green dots, those exhibiting a decrease in activity during hypoxia are represented as red dots. Note that most elements exhibited a decrease in firing. Blue dots represent elements whose firing switched off in the last minute of hypoxia. The histogram on the right shows that mean firing frequency in control conditions (black and gray bars) and during hypoxia (white bars) for each class of element: unitary, multi-unitary, or merged (^*^p < 0.05).

**Figure 3:**
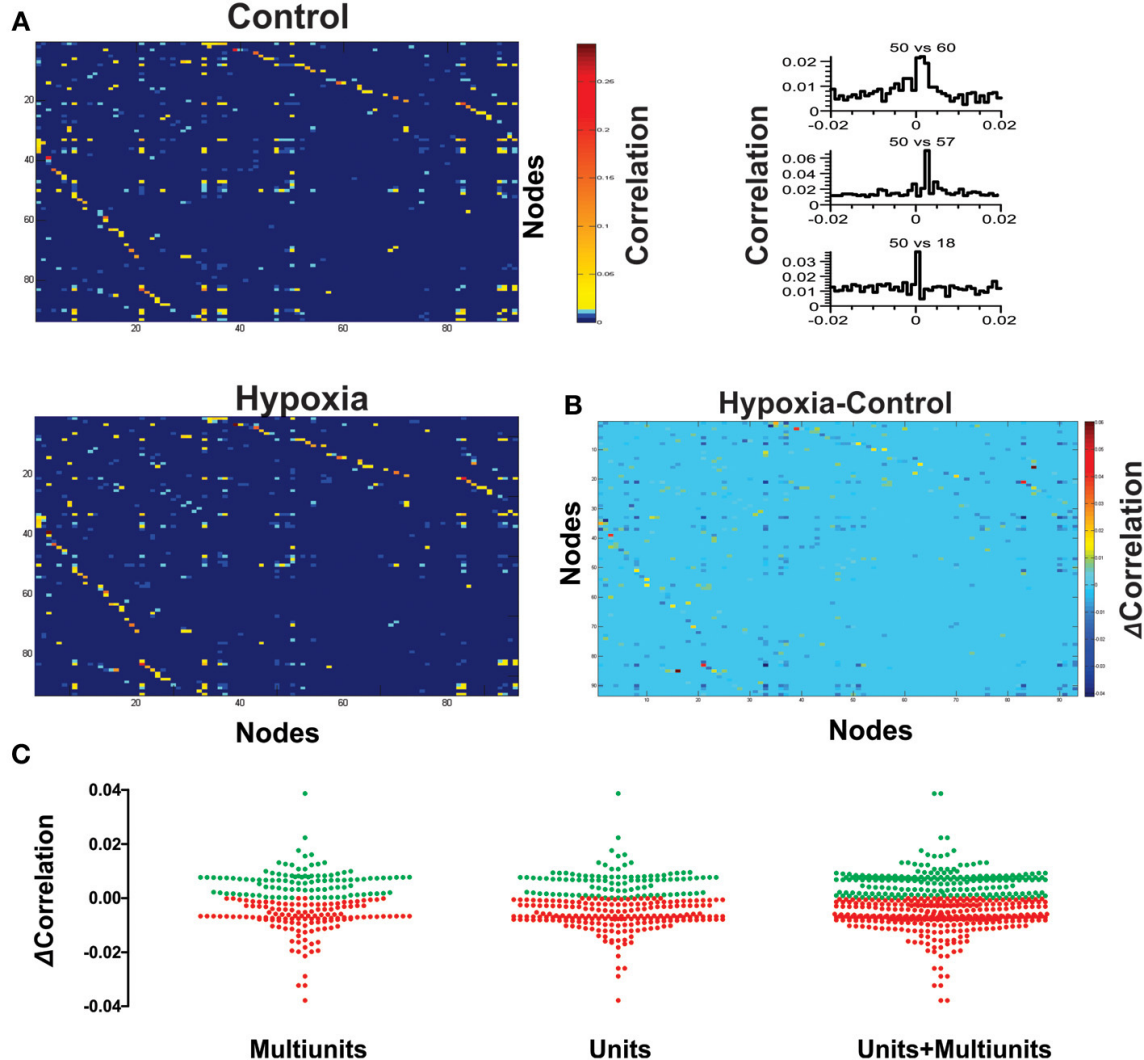
**Hypoxia reconfigures the functional interactions among the respiratory elements. (A)** Examples of cross-correlograms of the activity recorded between elements are presented on the right. All significant pair-wise-possible correlations are represented as correlation matrices on the left for a representative slice in control and during hypoxic conditions; a pseudocolored scale indicates that different pairs are correlated to different extents. **(B)** Subtraction of correlation values of the matrices represented in **(A)**. Color scale indicates the extent of change of correlation values (Δ Correlation). Note that the correlation increases for a few pairs of respiratory elements, but it decreases for most of them. **(C)** The graph shows a pair to pair change in correlation under hypoxia conditions (ΔCorrelation) represented in the matrix of panel **(B)**. Data are grouped as unitary, multi-unitary, or both. Increase in correlation is denoted by green dots, decrease by red dots.

To evaluate the population burst pattern of preBötC activity we measured the peak-to-peak amplitude of the raw signal every 45 ms. By averaging the amplitude signals for several bursts, it is possible to clearly distinguish the changes in burst pattern occurring during the transition from fictive-eupnea to fictive-gasping (Figure 1A, inset; Lieske et al., 2000; Lieske and Ramirez, 2003; Peña et al., 2008) Recordings were pass-band filtered (250–7000 Hz) with MC_Rack Software (Multi-Channel Systems, Reutlingen, Germany). The channels exhibiting respiratory activity were selected (an average of 25 channels covering an area of 400 by 400 μm; Figure 1A; Carroll and Ramirez, 2013). Filtered channels containing the high frequency component of neural activity were exported to an OFFline Sorter program (v. 3.3.1; Plexon Inc., USA; Carroll and Ramirez, 2013). Files of those recordings were merged using PlexUtil program (v. 4.0.1; Plexon Inc., USA) with the last 10 min of isocitrate and/or hypoxia recordings merged to 10 min of control recordings. Back in OFFline Sorter, spikes were detected by setting a threshold 2.6 *SD* of the signal (Supér and Roelfsema, 2005; Galan et al., 2010), and individual units were distinguished from biological and electrical noise through principle component analysis (PCA) of the spike waveform patterns (Figure 1B; Supér and Roelfsema, 2005; Galan et al., 2010), with a semi-automatic approach using the standard expectation-maximization algorithm from OFFline sorter. When rhythmic spiking activity could not be classified as coming from individual respiratory units (unitary), we recorded MUA produced by a cluster or group of neurons (Bedenbaugh and Gerstein, 1997; Supér and Roelfsema, 2005). In such a case, to decrease the noise in multiunit activity, the threshold for spike detection was raised to 4.0 *SD* of the signal. Spikes that occurred within the refractory period (set as 2 ms) of the selected units or multiunits (always less than 0.5%) were discarded (Figure 1B). The sorting was verified by the existence of a refractory period in the interspike interval histogram (2 ms), as well as auto- and cross-correlation histograms examined with Neuroexplorer (v. 4.126, Nex Technologies, USA; Figure 1B), as a check on sorting results (Bedenbaugh and Gerstein, 1997; Supér and Roelfsema, 2005). Timestamps of unitary and multiunitary recordings were used to build raster plots (Figure 2A), which were exported to MATLAB (version R2011b) to be further analyzed with custom-made routines. Detected spikes were converted to binary times of occurrence with a 1-ms bin (Figures 1B, 2A). The quasi-simultaneous occurrence of action potentials among pairs of respiratory units and/or multiunits was assessed by means of cross-correlation analysis (Figure 3A; Perkel et al., 1967; Kirkwood, 1979; Kashiwagi et al., 1993; Bedenbaugh and Gerstein, 1997; Shen et al., 2002; Li et al., 2003; Supér and Roelfsema, 2005; Eugenin et al., 2006; Segers et al., 2008; Ott et al., 2011; Lalley and Mifflin, 2012; Road et al., 2013) with a lag window of ± 5 ms (Figure 3A). Autocorrelation and cross-correlation functions were normalized to the firing rate to assure that any change in correlation values was independent of changes in firing frequency (Nini et al., 1995; Heimer et al., 2002). Cross-correlations were considered significant when the correlation peak reached values >5 *SD* of the correlation noise (>99.9% confidence interval). We calculated the mean frequency of recordings for both units and multiunits. The reciprocal of the median inter-spike intervals (ISI) was taken to obtain an approximation of intraburst frequency of inspiratory neurons (Galan et al., 2010). Our analysis included both inspiratory and expiratory neurons (or non-inspiratory neurons). Correlation linkage matrices were built for each slice in each experimental condition (Figures 3B, **5A**; Segers et al., 2008; Ott et al., 2011). The matrices contain the correlation value of those interactions that reached the significance threshold (5 *SD* of correlation noise); correlations that did not reach this threshold received a value of zero (Figures 3A, **5A**). To compare the strength of the correlations in the presence of hypoxia and/or isocitrate, we subtracted the control correlation matrix from the correlation matrices in any given experimental conditions and used the resulting values as the change in correlation (ΔCorrelation) (Figures 3B, **5B**). Similarly, the correlation matrices in the presence of isocitrate were subtracted from the control matrices in hypoxic conditions in the presence of isocitrate. The graphic representation of the network in any given condition was made with the open access software Cystoscape (Cytoscape Consortium).

All quantifications were grouped by unitary, multi-unitary, or the merge of both. Representative data are given as mean ± s.e.m., and the median frequency is reported with the interquartile range. Statistical differences among groups were tested using either a paired or unpaired Student's *t*-test or Repeated Measures ANOVA followed by a *post-hoc* pair-wise Tukey's or Dunnett's test as needed using GraphPad Prism and, in a few cases, (as for median frequency) Kruskal-Wallis ANOVA followed by the Dunn's test. Statistical significance was accepted at p-values <0.05.

## Results

### Changes in the respiratory network during hypoxia

Brainstem slices were placed on top of 6 × 10 MEA arrays (Figure 1A) and recorded in an area including the preBötC. They show the typical fictive-eupnea activity generated by the preBötC in control conditions and the change in burst pattern that characterizes the transition to fictive-gasping (fewer, shorter, sudden, and decreasing bursts; Lieske et al., 2000; Peña, 2009) in hypoxia (Figure 1A, inset). From the MEA recordings of 12 slices, a total of 427 respiratory units (17 to 59 per slice; Figure 1B) and 322 multi-unitary recordings (13–44 per slice; Hartelt et al., 2008; Mironov, 2009) were included. Thus, a total of 560,252 pair-wise cross-correlations were performed, and 0.5% of those correlations exhibited significant peaks with values 5 *SD* above the correlation noise (confidence interval > 99.9%) and within a lag period of ± 5 ms. As expected, respiratory multiunitary recordings exhibited inspiratory activity. Of the unitary recordings, 87% were also identified as inspiratory units (range: 73–100%) (Figures 1C, 2A). The rest of the respiratory units (13%; range: 0–27%) were cataloged as expiratory or non-inspiratory units (Figures 1C, 2A).

**Figure 4:**
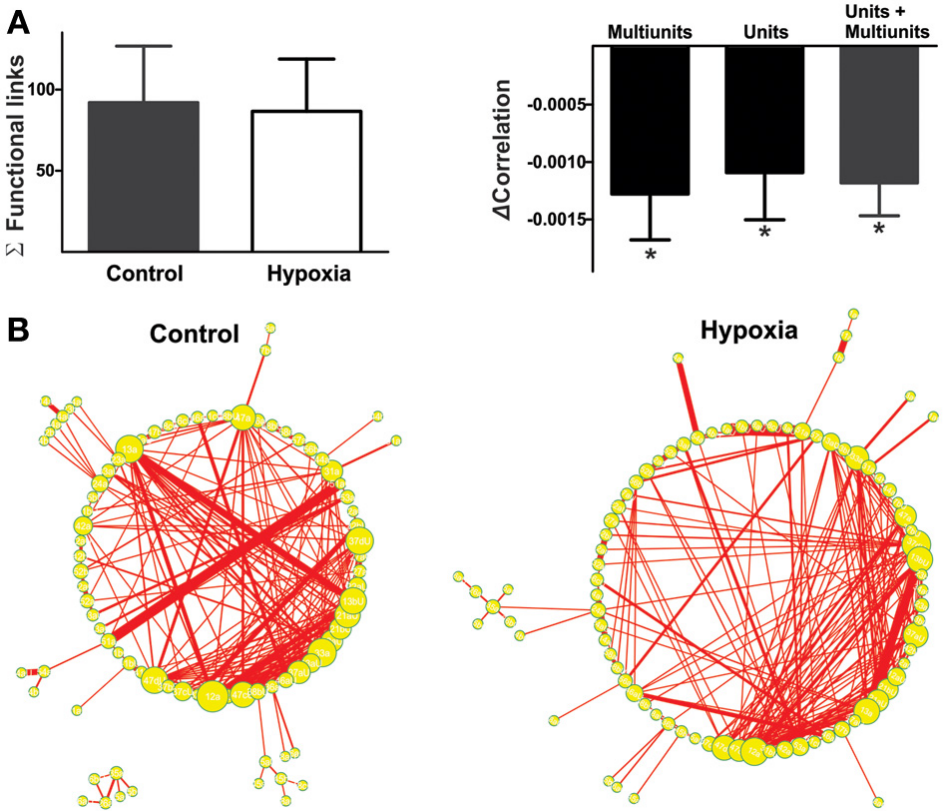
**The reconfiguration of the respiratory network in hypoxia does not imply a loss of functional links but a reduction in their strength**. **(A)** Left: Histogram of the number of links in the respiratory network, indicating functional connectivity between elements did not change significantly with hypoxia. Right: Histogram of the change in correlated firing among elements between normoxic and hypoxic conditions, showing a significant reduction in correlation for all classes of respiratory recording elements: multi-unitary, unitary and both (^*^p < 0.05). **(B)** Graphic representation of respiratory network configurations in normoxic and hypoxic conditions. Each respiratory element is represented as a circle and each significant correlation between two elements is represented as a connecting line (functional link). The diameter of the circles is proportional to the number of functional links that each element has with other elements in the network. The width of the line is proportional to the correlation value of a given link. Note that the reconfiguration of the respiratory network in hypoxia is not due to a loss in the number of respiratory elements or of their functional links, but to a reduction in the strength of such links.

Representative raster plots of the preBötC activity are displayed in Figure 2A in control conditions (Figure 2A, left) and at the end of the hypoxic period (15 min; Figure 2A, right). Each row in the raster plots represents the activity obtained from a respiratory element recorded from an electrode of the MEA. Rows include both unitary (bottom) and multi-unitary (top) recordings (Figure 2A). From these activity rasters and from the quantification of the firing frequency, it can be observed that the transition from fictive-eupnea to fictive-gasping activity involves diverse, and even opposed, changes in firing frequency (Figure 2) for different elements recorded by the electrodes of the MEA. A subset of neurons clearly increase their firing frequency to 135 ± 6% (mean ± s.e.m.) of the control (Figures 2B, 4), whereas another subset of neurons decrease their firing rate to 64 ± 2% of the control (Figure 2B). However, a larger proportion (66%) of the neurons decrease their firing rate. Globally, the average firing rate in the circuit decreases in hypoxia to 88 ± 3% of the control (*p* < 0.05; Figure 2B).

To approximate the change in intraburst frequency in hypoxia (Galan et al., 2010), we measured the inverse of the median interspike interval, which had a median value in control conditions of 5.26 Hz (interquartile range: 1.72–12.82 Hz) and was significantly reduced to 3.81 Hz in hypoxia (interquartile range: 1.16–11.43 Hz). Surprisingly, very few elements (0.4%) of the net stopped firing (Figure 2B; blue dots). These results contrast with previous suggestions by cell-focused studies, using patch clamp recordings in synaptically isolated neurons (Peña, 2009). Therefore, as a first conclusion, hypoxia shuts down the activity of very few respiratory neurons; on the contrary, it may increase the firing frequency of many of them. In general, however, the dominant tendency is a decreased firing frequency in most recorded elements and thus, reduced global network activity (Figure 2B).

Next, we proceeded to characterize network interactions by means of cross-correlation analysis among respiratory elements (Figure 3). Cross-correlograms obtained from pairwise analysis between elements in a given slice exhibit a wide variety of shapes (Figure 3A, right): peaks that included the zero lag (38% for the network shown in Figure 3A), peaks with lags between 0.1 and 2 ms (37% for the network shown in Figure 3A), and peaks with lags between 2.1 and 5.0 ms (25% for the network shown in Figure 3A). Of the correlograms that exhibited a peak at zero, some were broad peaks (25%) and the rest were sharp peaks (75% of the network shown in Figure 3A). As already mentioned, only correlations with peaks > 5 *SD* of the noise correlation were considered functional connections and included in correlation linkage matrices. In general, the larger the correlation values above this threshold, the stronger we considered the strength of the functional connections between the elements recorded by the electrodes of the MEA to be (Figure 3A). Correlation linkage matrices reveal that the strength of the interactions among respiratory elements is quite diverse (Figure 3A). Moreover, such interactions among respiratory elements change during the reconfiguration of the respiratory network in hypoxia (Figure 3). While the correlation value of some links increased during hypoxia, in most links it decreased (Figures 3B,C), suggesting that the strength of network interaction is diminished (Figures 3C, 4A). The averaged change in correlation (ΔCorrelation) was significantly negative for units, multiunits, and both (Figure 4A). Despite this reduction in the strength of the functional links among elements in hypoxic conditions, the actual number of functional links in the network remained unaltered in hypoxia (92 ± 35 in normoxia and 87 ± 33 in hypoxia Figure 4A; NS, *N* = 5 slices). Figure 4B illustrates the configuration of the respiratory network both in normoxic and hypoxic conditions. Elements are denoted by circles and functional links by lines; the larger the circle, the more connections it has. Thicker lines mean more correlated firing between the elements involved. In summary, the respiratory circuit exhibits a significant decrease in overall activity and a significant parallel decrease in the strength of functional connectivity, but neither the number of active neurons nor the number of functional connections changed significantly (Figure 4B).

### Actions of isocitrate during normoxia and hypoxia

**Figure 5:**
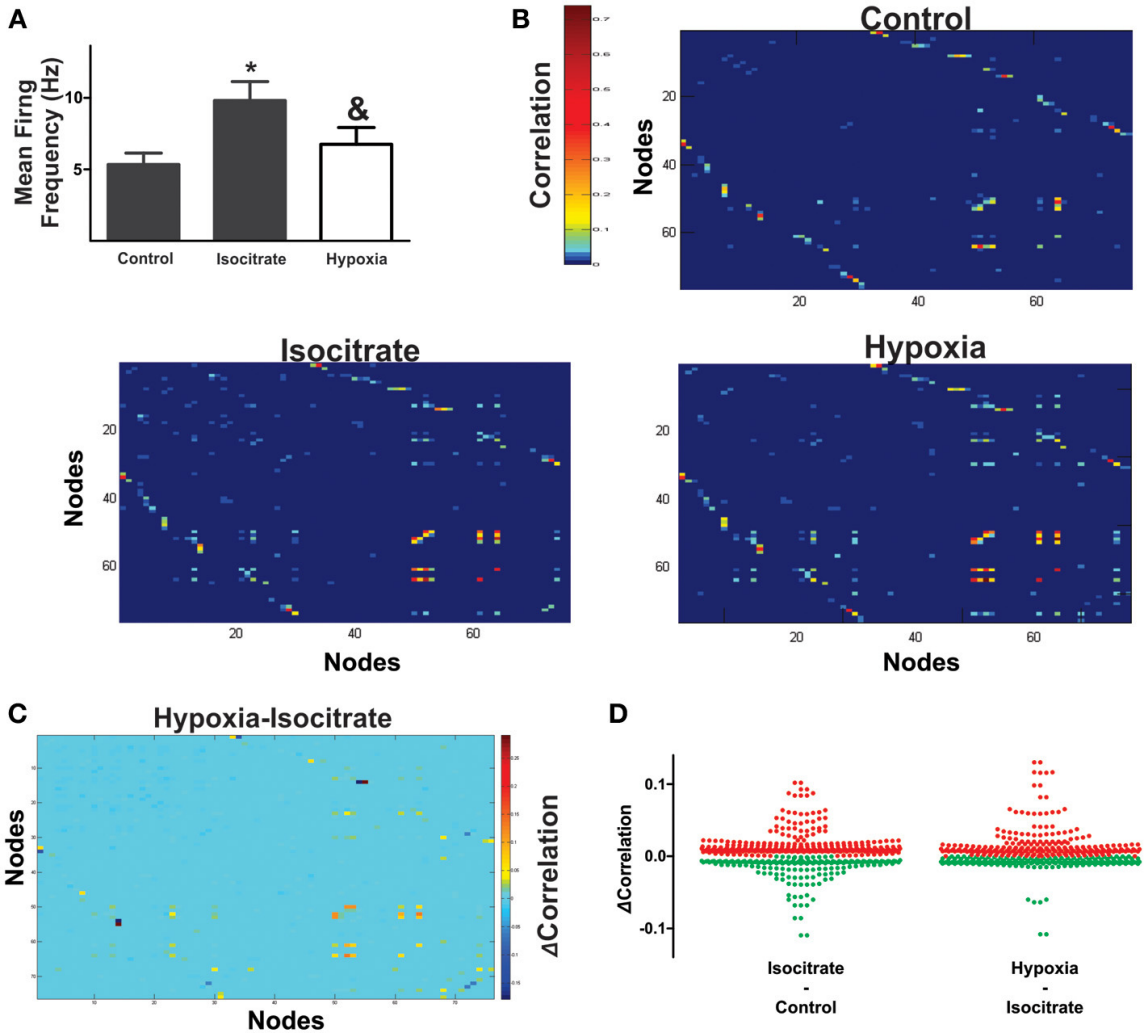
**Isocitrate increases functional interactions among respiratory elements and increases correlated firing in hypoxia**. **(A)** Histogram of the normalized mean firing frequency recorded in the control, during the last minute of isocitrate application (which lasted 60 min) and under hypoxic conditions in the presence of isocitrate (which lasted 15 min; *n* = 7 slices from different animals). ^*^Denotes a difference with respect to control, and & denotes a difference with respect to isocitrate (*p* < 0.05). **(B)** Pseudocolored correlation matrices between all pairs of recording elements in a representative slice (control) and in the same slice in the presence of isocitrate and further application of hypoxia. Color scale indicates the extent of correlation. Note more correlated element pairs after isocitrate and during hypoxia in the continuous presence of isocitrate. **(C)** Differences in correlation values for all recording element pairs were assessed by subtracting correlation values during hypoxia from those with isocitrate. Color scale denotes change in correlation (ΔCorrelation). Note that for a few pairs of respiratory elements, the correlation decreased (blue), while it increased for most pairs (hot colors). **(D)** The plots show changes in correlation of all paired comparisons between elements: first, after adding isocitrate in normoxic conditions and then, in the presence of isocitrate in hypoxic conditions (multi-unitary and unitary recordings were added together; increased correlations are denoted by red dots, decreased correlations are denoted by green dots). Note that most element pairs increase correlated firing when isocitrate is present in hypoxic conditions.

As previously reported (Hülsmann et al., 2000; Rivera-Angulo and Peña-Ortega, 2014), isocitrate has an excitatory effect on the respiratory network. Upon isocitrate application during normoxia, most of the respiratory elements (84%) registered increases in firing frequency to 463 ± 45% of the control, whereas another small subset of elements recorded decreases in firing rate to 58 ± 38% of the control (Figure 5A). Intraburst firing frequency exhibited a median of 2.39 Hz (interquartile range: 0.85–10.36 Hz) in control conditions and was significantly increased by isocitrate to 4.57 Hz (interquartile range: 1.89–15.15 Hz).

**Figure 6:**
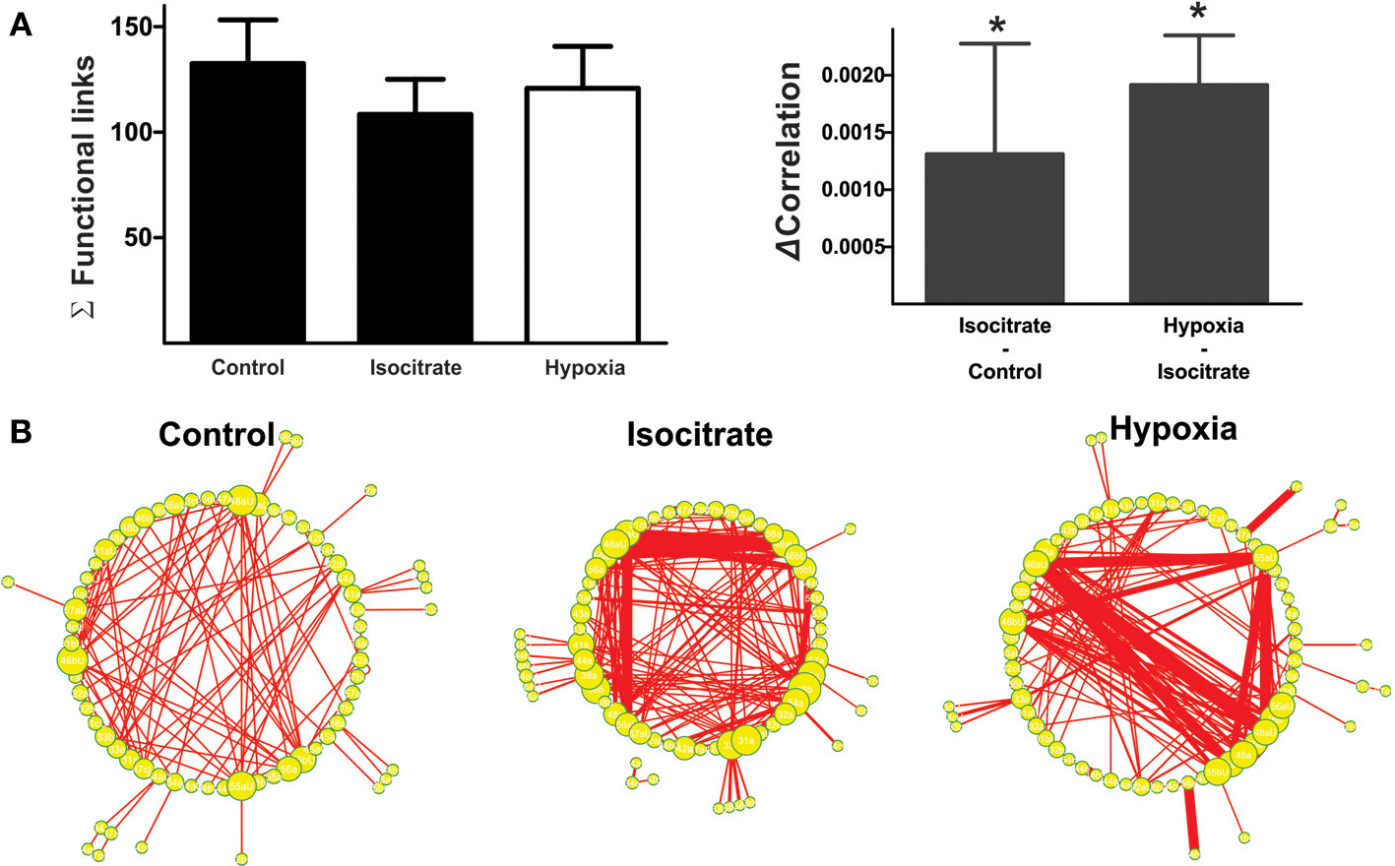
**Isocitrate reconfigures the respiratory network by changing firing frequency of respiratory elements and connectivity strength between them. (A)** Left: Histogram of the number of links in the respiratory network, indicating that the functional connectivity between elements did not change significantly in the presence of isocitrate and further application of hypoxia. Right: Histogram of the change in correlated firing among elements between normoxic and hypoxic conditions (multiunits and units were grouped together), showing a significant increase in correlation in the presence of isocitrate and a further increase upon hypoxia application (^*^p < 0.05). **(B)** Graphic representation of respiratory network configurations in normoxia before and after isocitrate application and in hypoxic conditions in the presence of isocitrate. Each respiratory element is represented as a circle and each significant correlation between two elements is represented as a connecting line (functional link). The diameter of the circles is proportional to the number of functional links that each element has with other elements in the network. The width of the lines is proportional to the correlation value of any given link. Note that isocitrate increases the strength of the functional links but does not significantly change their number. When hypoxia is applied in the presence of isocitrate, the strength of the functional links increases even further, but again, their number remains constant.

Correlation matrices show that isocitrate increases the strength of most of the functional links among respiratory elements (Figures 5B–D). Thus, the averaged change in correlation (ΔCorrelation) was significantly positive upon isocitrate application (Figure 6A). Despite this increase in the strength of the functional links among elements upon isocitrate application, the actual number of functional links in the network remained unaltered (133 ± 21 in normoxia and 108 ± 18 in isocitrate, Figure 6A; NS; *n* = 7 slices; *p* > 0.05 by means of ANOVA). Figure 6B illustrates the change in respiratory network configuration in the presence of isocitrate, showing that isocitrate increases not only the activity of the respiratory elements (Figure 5A) but also the strength of the functional links in the circuit in normoxic conditions. A question is whether isocitrate can prevent the loss of activity and sustain strong functional connectivity in case hypoxia supervenes (since its clinical action would be to prevent vulnerable children from entering into the hypoxic state; Peña and García, 2006). To answer this, we then subjected the isocitrate-treated tissue to hypoxic conditions (Figures 5, 6). It was seen that during hypoxia firing significantly decreased, returning to control levels (Figure 5A). Intraburst frequency was reduced to a median of 2.47 Hz (interquartile range: 1.05–8.99 Hz), which is not significantly different from the median firing frequency before isocitrate application (2.39 Hz) but was significantly smaller than in the presence of isocitrate in normoxia (4.57 Hz). In spite of the reduction in firing frequency in hypoxia, isocitrate increased the strength of functional links in hypoxic conditions (Figures 5B–D, 6A). Since the correlation value of most functional links among respiratory elements increased when hypoxia was applied in the presence of isocitrate (Figures 5C,D), the averaged change in correlation (ΔCorrelation) was a significant positive value when compared with its normoxic value in the presence of isocitrate (Figure 6A). Although recorded elements of the respiratory network show a decreased firing rate in hypoxic conditions in the presence of isocitrate (Figure 5A), the strength of the functional links of the circuit is increased as compared with normoxic conditions in the presence of isocitrate (Figure 6B). Note that this is contrary to what happens in slices in the absence of isocitrate (Figure 4B).

## Discussion

The reconfiguration of the respiratory CPG that transforms the preBötC network from a control configuration that generates fictive-eupnea in normoxia to a hypoxic configuration that generates fictive-gasping involves complex changes at single-cell and network levels (Ramirez et al., 2007, 2013; Peña, 2009). Here, we studied this reconfiguration by MEA recordings and analyzed three possible contributions to such a change: changes either in the number of respiratory elements recorded by the MEA electrodes, changes in the functional connections among elements, and/or changes in the strength of these connections assessed by their correlated firing. We found that the reconfiguration of the respiratory network in hypoxia does not involve a significant change in the number of respiratory elements participating in network dynamics (Figure 3) or in the number of functional links among them (Figure 4). Instead, it involves changes in firing frequency and in the strength of functional connectivity among network elements (Figures 3–5). Moreover, we found that the metabolic intermediate isocitrate, by itself, changes the respiratory network configuration, increasing the strength of functional connectivity even in normoxia thereby preventing the loss of this strength during hypoxia, so that the circuit does not reach the low levels of functional connectivity found in untreated hypoxic slices. Isocitrate also increases the average firing rate of the recorded elements (Figure 5A).

Changes in respiratory network configuration leading to gasping generation have both basic and clinical implications. On the one hand, it might represent the best example of an acute and reversible network reconfiguration in a mammalian CPG (Ramirez et al., 2007; Peña, 2009), and on the other hand, understanding the cellular basis of gasping generation would help to understand SIDS pathophysiology, since babies who die from SIDS have defects in the mechanisms generating gasping and autoresuscitation (Lijowska et al., 1985; Poets et al., 1999).

Since the seminal study by Lieske et al. (2000), it has been shown that the CPG located in the preBötC is not a fixed network, but rather a highly flexible, neural circuit that can change its configuration, functionally adapting to the metabolic demands that several physiological and pathological conditions impose on breathing generation (Peña and Ramirez, 2005; Ramirez et al., 2007; Mironov, 2009; Peña, 2009; Galan et al., 2010). Reconfiguration of the respiratory network under hypoxic conditions was proposed many years ago (Lieske et al., 2000). However, other than inferences obtained by cell-focused studies (Richter et al., 1991; Ballanyi et al., 1994; Ramirez et al., 1998; Lieske et al., 2000; Thoby-Brisson and Ramirez, 2000; Peña et al., 2004; Paton et al., 2006; Zavala-Tecuapetla et al., 2008; Ramírez-Jarquín et al., 2012), there was almost no ways to globally assess circuit configuration of the preBötC in both conditions (normoxia and hypoxia). Consistent with our findings, previous studies predicted that hypoxia produces a heterogeneous effect on both the intrinsic (St. John and Bianchi, 1985; Richter et al., 1991; Ballanyi et al., 1994; England et al., 1995; Ramirez et al., 1998; St. John, 1999; Lieske et al., 2000; Thoby-Brisson and Ramirez, 2000; Peña et al., 2004; Paton et al., 2006; Zavala-Tecuapetla et al., 2008) and the synaptic properties (Richter et al., 1991; Ballanyi et al., 1994; Ramirez et al., 1998; Lieske et al., 2000; Thoby-Brisson and Ramirez, 2000) of respiratory neurons. However, whereas some cell-focused studies reported that hypoxia depressed the firing of large subsets of respiratory neurons (St. John and Bianchi, 1985; Richter et al., 1991; Ballanyi et al., 1994; England et al., 1995; St. John, 1999; Thoby-Brisson and Ramirez, 2000) others showed that hypoxia increased (Richter et al., 1991; Lovering et al., 2006) or did not change the firing of neurons within the respiratory network (Lieske et al., 2000; Thoby-Brisson and Ramirez, 2000; Lovering et al., 2006). Regarding synaptic transmission, the most consistent observation is that hypoxia depressed synaptic inhibition within the preBötC (Richter et al., 1991; Ballanyi et al., 1994; England et al., 1995; Schmidt et al., 1995; Ramirez et al., 1998; Lieske et al., 2000; Thoby-Brisson and Ramirez, 2000), and in agreement with the present findings, the effects of hypoxia on synaptic excitation were diverse (Ballanyi et al., 1994; Ramirez et al., 1998). Using a network approach we could determine the suspected heterogeneity of the effects of hypoxia on the preBötC. For instance, the change in firing frequency upon hypoxia showed both elements that decreased and others that increased their firing frequency (St. John and Bianchi, 1985; Richter et al., 1991; Ballanyi et al., 1994; England et al., 1995; St. John, 1999; Lieske et al., 2000; Thoby-Brisson and Ramirez, 2000; Lovering et al., 2006). Nevertheless, we did not observe the switching off of many neurons, which had been suspected from cell-focused approaches (St. John and Bianchi, 1985; Richter et al., 1991; Ballanyi et al., 1994; England et al., 1995; St. John, 1999; Thoby-Brisson and Ramirez, 2000) and was recently incorporated into the current model of the network reconfiguration of the preBötC in hypoxia (Ramirez et al., 2007; Peña, 2009; Garcia et al., 2013). Using functional connectivity, as is done in systems neurophysiology (Lindsey et al., 2000; Segers et al., 2008; Ott et al., 2011), we also observed the predicted heterogeneity in the changes of connections and interactions during hypoxic conditions (Ballanyi et al., 1994; Ramirez et al., 1998) and provided quantitative evidence that these changes in functional coupling, and not a change in the number of respiratory elements or the number of functional links among them, seem to be a major component of the respiratory network reconfiguration in hypoxia.

Here we considered as “respiratory recording nodes” all electrodes in the array capable of recording both unitary and multi-unitary activity (Bedenbaugh and Gerstein, 1997; Supér and Roelfsema, 2005), as shown in different brain regions (Bedenbaugh and Gerstein, 1997; Supér and Roelfsema, 2005; Segers et al., 2008; Morris et al., 2010; Lalley and Mifflin, 2012; Road et al., 2013). Anatomical imaging studies have shown that the preBötC is constituted of clusters of highly connected respiratory neurons (Hartelt et al., 2008; Mironov, 2009); thus, multi-unitary respiratory activity is a logical outcome for the recording nodes in the MEA. However, including MUA in a network analysis may require some caution (Bedenbaugh and Gerstein, 1997; Supér and Roelfsema, 2005). For example, the changes in firing rate of individual neurons constituting a given MUA would not be perfectly reflected by the MUA firing rate if different neurons within the cluster have simultaneous but opposing effects on the firing rate (Bedenbaugh and Gerstein, 1997; Supér and Roelfsema, 2005). Additionally, the source of functional interactions (measured as a significant cross-correlation) among MUAs, or between a MUA and unitary activity, or the change in such interactions in a given experimental condition, can never be assigned to a specific pair of cells within the MUAs or to a specific cell when a MUA is correlated with a well-defined unit (Bedenbaugh and Gerstein, 1997; Supér and Roelfsema, 2005). In fact, a change in a correlation involving a MUA due to an experimental condition could just reflect a change in the composition of the neurons contributing to the MUA in this condition (Bedenbaugh and Gerstein, 1997; Supér and Roelfsema, 2005). Analyses including respiratory MUAs have provided relevant information in the past (Segers et al., 2008; Morris et al., 2010; Lalley and Mifflin, 2012; Road et al., 2013), and the global changes reported in this study were similar whether they were quantified from unitary, multi-unitary recordings, or the merge of both (Figures 2–4). Importantly, we evaluated the respiratory network configuration including interactions that occur within 5 ms (Perkel et al., 1967). The functional connections inducing respiratory network dynamics could be made either by synaptic connections among the recorded units or by using a shared common source to activate these units (Perkel et al., 1967; Kashiwagi et al., 1993; Onimaru et al., 1993; Li et al., 2003; Ott et al., 2011). Further research would dissect the nature of these interactions and include them in a broader framework (Carrillo-Reid et al., 2008; Jaidar et al., 2010; Peña et al., 2010).

In this study we have determined the global changes occurring in the respiratory network configuration under hypoxic conditions by analyzing the changes in cross-correlation among the elements recorded by the “recording respiratory nodes” of a MEA, as previously done in different neural networks (Galan et al., 2010; Gerhard et al., 2011), including the respiratory network (Galan et al., 2010). As far as we know, this is the first global description of such changes during the hypoxic conditions, when actual fictive-gasping activity is being generated. Our main finding is that the reconfiguration of the respiratory network in hypoxia mainly consists of a reduction in the strength of network connectivity rather than a loss, proposed earlier, of respiratory elements (Ramirez et al., 2007; Peña, 2009; Garcia et al., 2013). The observed re-arrangements of network interactions among the respiratory elements is consistent with a previous report that a brief application of cyanide (chemical hypoxia) induced the retraction of neuronal processes of respiratory neurons, which was interpreted as a reduction in connectivity among respiratory neurons (Mironov, 2009). Moreover, this change in the strength of interactions within the respiratory network in hypoxia can be related to the uncoupling of preBötC activity from one of its motor outputs (the hypoglossal nucleus) during fictive-gasping generation (Ramirez et al., 1998; Peña et al., 2008). However, the activity of the phrenic nerve is also uncoupled from hypoglossal nerve activity in hypoxia, which may explain why, under hypoxic conditions, the amplitude of the phrenic output is not reduced (St-John et al., 2004; St. John and Leiter, 2009). Thus, there are two possible explanations for the fact that the amplitude of phrenic output is maintained, or even increases, in hypoxia, despite the reduced connectivity within the respiratory rhythm generator: First, although preBötC bursts are certainly shorter than those in normoxia, their amplitude is not reduced (Figure 1A, inset; Peña et al., 2008). Second, in more intact conditions, modulations provided by respiratory circuits beyond the preBötC could help to maintain or even increase the phrenic output amplitude in hypoxia (St-John et al., 2004; St. John and Leiter, 2009). Our finding that the preBötC is able to change its population burst pattern by just changing the strength of the interactions among its elements contributes to the proposal that CPGs are not simple hardwired networks that produce simple behaviors but, on the contrary, they constitute flexible circuits that can be reconfigured in response to the environmental, behavioral, and metabolic states of the animal (Marder, 1994). These reconfigurations include changes in the number of elements, the number of functional links, and/or the strength of these connections (Marder, 1994). More experimental testing and modeling will be required to determine whether or not the changes reported in this study are necessary and sufficient to induce the respiratory pattern change that occurs in the transition from normoxia to hypoxia.

The other major finding of the present study is that isocitrate can change the preBötC configuration, which would explain why isocitrate promotes eupnea and gasping generation both *in vivo* and *in vitro* (Hülsmann et al., 2000; Rivera-Angulo and Peña-Ortega, 2014) and suggests that it be used as a preventative strategy to oppose some of the changes in network configuration that occur in hypoxic conditions. It is well known that the respiratory network is very efficient in anaerobic metabolism (Ballanyi et al., 1996), and that this might be the reason why an intermediate of the Krebs cycle would potentiate the activity of the respiratory network under metabolic distress (Hülsmann et al., 2000; Rivera-Angulo and Peña-Ortega, 2014). The beneficial effects of isocitrate were observed here as an increase in the strength of network interactions in normoxia and as an increase in neuronal activity; moreover, isocitrate prevented the loss of these interactions and activity during the transition to fictive-gasping generation in hypoxia. These observations agree with the finding that application of several metabolic intermediates increases spontaneous network activity in other brain circuits by modulating action potential generation as well as synaptic transmission (Perasso et al., 2008; Gavello et al., 2012; Garbati et al., 2014). Our findings are also consistent with previous reports that, in several brain areas including the respiratory network (Dehaven and Carpenter, 1964; Böhmer et al., 1976; Chaplain et al., 1976; Dinse et al., 1976), different metabolic intermediates modulate neuronal firing and synaptic transmission (Shoji, 1992; Perasso et al., 2008; Gavello et al., 2012; Garbati et al., 2014). Nevertheless, to the best of our knowledge, this is the first evidence that a metabolic intermediate induces changes in network configuration parameters (Figure 6). Moreover, our data suggest that pharmacological manipulations that increase both respiratory neuron firing rates and the functional interactions among respiratory elements are potential pharmacological tools to promote gasping generation and autoresuscitation (Peña and García, 2006; Peña, 2009), which would have a beneficial impact in those babies at risk for SIDS (Peña and García, 2006; Peña, 2009; Ramirez et al., 2013).

### Conflict of interest statement

The authors declare that the research was conducted in the absence of any commercial or financial relationships that could be construed as a potential conflict of interest.
